# Three-dimensional minimally invasive video-assisted thyroidectomy: preliminary report

**DOI:** 10.1186/1756-9966-32-78

**Published:** 2013-10-18

**Authors:** Giuseppe Mercante, Paolo Battaglia, Valentina Manciocco, Giovanni Cristalli, Raul Pellini, Giuseppe Spriano

**Affiliations:** 1 Department of Otolaryngology-Head and Neck Surgery, Regina Elena National Cancer Institute Rome, Via Elio Chianesi 53, 00144 Rome, Italy

**Keywords:** MIVAT, 3D, Three dimensional, Minimally invasive, Thyroidectomy

## Abstract

Three-dimensional (3D) minimally invasive video-assisted thyroidectomy (MIVAT) was carried out with a 4-mm, 3D 0-degree stereoscopic endoscope. The procedure was applied on 3 patients who underwent total thyroidectomy and data were prospectively collected. Operative time for total thyroidectomy ranged from 72 to 90 minutes. Neither intra-nor post-operative complications were reported during the study.

The surgical team noticed a good perception of depth and easy recognising of anatomic structures, especially concerning the upper and lower vascular pedicle, the parathyroids, the superior and inferior laryngeal nerves. Preliminary impression emerging from this study seems to suggest that 3D MIVAT is safe and effective. Future studies with larger case series are required to determine the role of this procedure.

## Background

Minimally invasive video-assisted thyroidectomy (MIVAT), described in 2001 by Miccoli [1], is one of the preferred approaches used for <25-30 mL of volume thyroid. MIVAT is currently performed using 2-dimensional (2D) 30° 5 mm endoscopes that lack in stereoscopic vision and depth of field. The recent introduced 4 mm 3D-endoscopes seem to overcome these limits in various surgical fields, particularly skull base, paranasal sinuses and neuro-surgery.

The aim of this study was to investigate the safety and effectiveness of new 3D endoscopes applied for MIVAT procedure.

## Methods

### Patients

In June 2013, three patients with multinodular goiter were enrolled to undergo 3D MIVAT with miniature stereoscopic camera (Visionsense Ltd, Petach-Tikva, Israel). This study was approved by the Institutional Review Board of the National Cancer Institute Regina Elena of Rome. Inclusion criteria to be admitted into the study were: thyroid with dominant nodule less than 3 cm in diameter, thyroid gland volume less than 25 mL, as shown in the ultrasound, no previous neck surgery or irradiation. All patients underwent total thyroidectomy according to the technique described in literature [1].

### Technology

**Figure 1:**
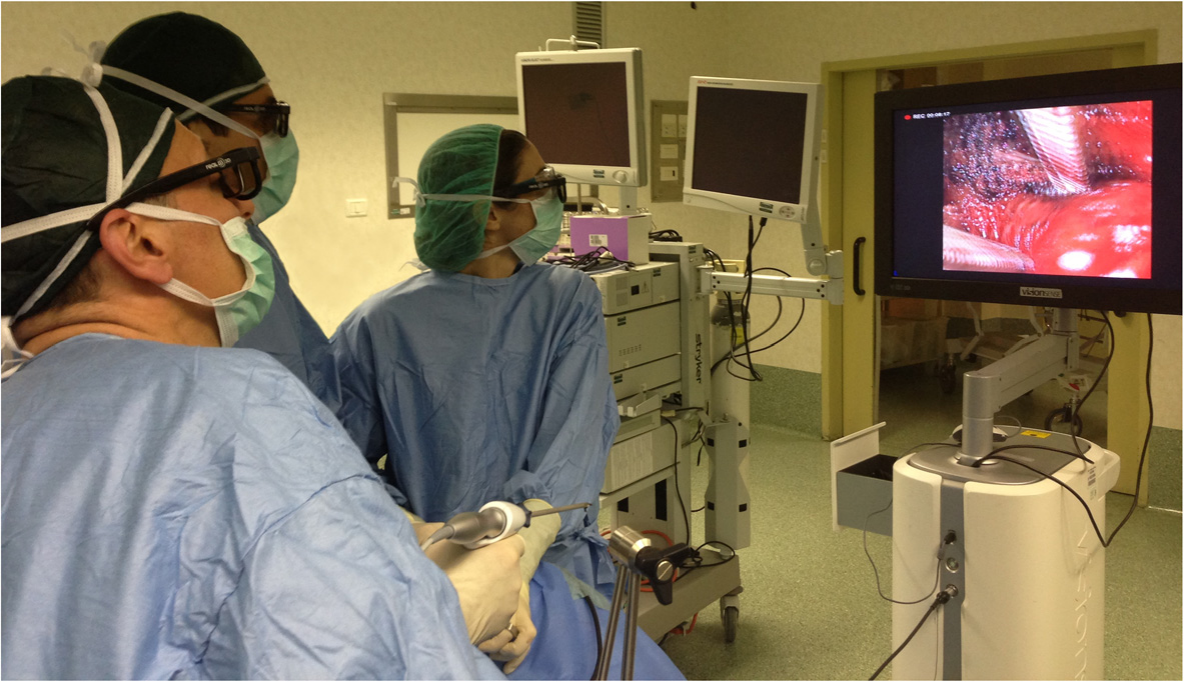
**Minimally invasive video-assisted thyroidectomy.** A view of the setting (endoscope, video camera and glasses) used for the 3D-MIVAT.

A 2 cm horizontal incision was made 1 cm below the inferior border of the cricoid cartilage, followed by the MIVAT technique [1]. A 4 mm, 3D 0-degree stereoscopic endoscope was used for the endoscopic part (Figure 1). The Visionsense endoscopic lens was adopted during all the procedure. It uses technology that incorporates a microscopic array of lenses (similar to an insect’s compound eye) in front of a single video chip on the end of the scope. Multiple small images are generated and then divided into simultaneous left and right images. Finally the viewer’s eyes simultaneously pick up two slightly different images of the same object.

### Assessment

Surgical team was composed by three surgeons trained in 2D MIVAT and with an experience of at least more than 30 MIVAT and 100 conventional thyroidectomies. Data were collected considering clinical and pathologic findings, technical aspects, operative time, rate of conversion into conventional technique and complications. The degree of difficulty of the 3D MIVAT technique was graded by the surgeon using a 5-point subjective scale, ranging from 5 (very easy) to 1 (very difficult) in order to recognize the upper and lower vascular pedicles, the parathyroids, the superior and inferior laryngeal nerves. Patients were asked to report their opinion according to the cosmetic results and postoperative pain. Cosmetic results were graded using a 4-point scale, ranging from 1 (very happy) to 4 (unhappy), while postoperative pain was evaluated by a visual analogue scale (VAS) from 1 (no pain ever) to 10 (worse pain).

## Results

**Figure 2:**
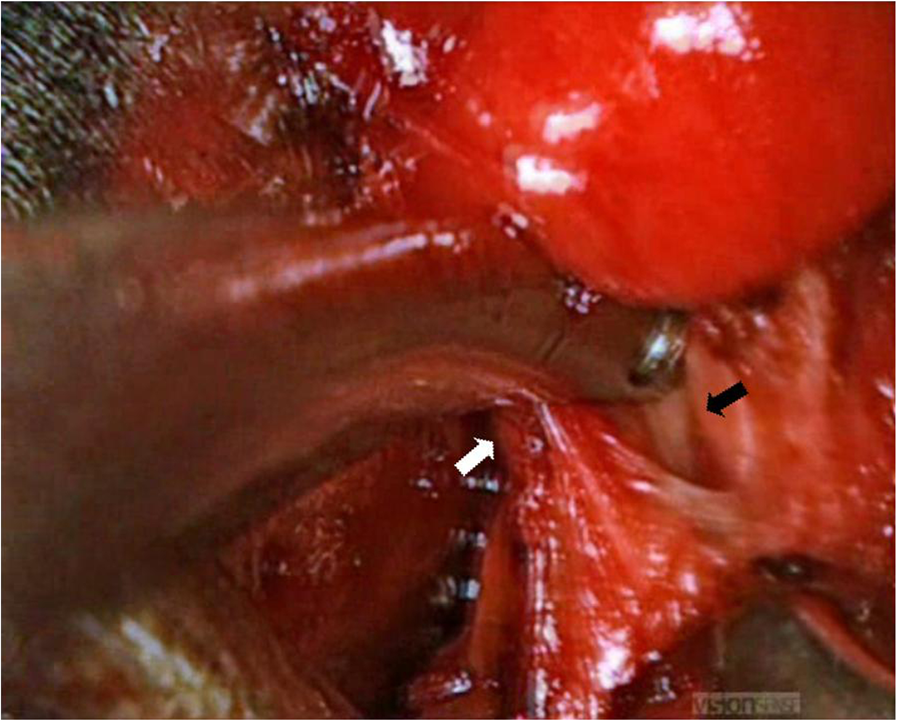
**An intraoperative 3D view of the operative field.** The upper vascular pedicle (white arrow) and the superior laryngeal nerve (black arrow) on the right side are easily recognized with good depth perception by the surgical team.

Two female and 1 male with a mean age (±SD) of 44.5 years (±8.4) underwent 3D MIVAT. Mean operative time for the total thyroidectomy was 80 minutes (range 72-90). Conversion into conventional technique was never required. Neither intra-nor postoperative complications were observed during the study. A suction drain was placed at the end of surgery and it was removed when blood loss was <2 mL/hour. All patients were discharged 24 hours after surgery. Table 1 summarizes clinical, pathologic and operative findings. The surgical team noticed a good perception of depth and easy recognising of anatomic structures, especially concerning the upper and lower vascular pedicles, the parathyroids, the superior and inferior laryngeal nerves (Figure 2). The recognition of these anatomic structures worsened in presence of blood in the surgical field. This new perception of depth and volume allowed an easy use of the endoscope during the procedure and an intuitive manipulation of critical structures, making comfortable and safe surgical maneuvres using instrumentation. The negligible weight of the handle and the absence of lateral cables made the device light and easy to manage. The surgeons wore polarizing glasses without any problem even during the open part of the surgery. No user side-effects related to the dual-camera device were reported. Two surgeons considered the technique as very easy, while one surgeon as easy. All patients were very happy about the cosmetic results. Pain VAS at 1, 3 and 7 postoperative day ranged from 1 to 2 in all cases. Table 2 summarizes the subjective qualitative evaluation of 3 D endoscopic system.

**Table 1:** Clinical, pathologic and operative findings of the patients

**Patients**	**Goiter volume (mL)**	**Dominant nodule major diameter (cm)**	**Operative time (min)**	**Intraoperative blood loss (mL)**	**Postoperative blood loss (mL)**	**Pathologic findings**	**Hositaliztion (days)**
**No. 1**	20	2.8	90	45	30	Follicular adenoma	1
**No. 2**	18	1.4	78	35	25	Multinodular goiter	1
**No. 3**	22	1.1	72	35	10	Multinodular goiter	1

**Table 2:** Subjective qualitative evaluation of 3D endoscope system

**Surgeons**	**Recognition of structures**					
	**Upper vascular pedicle**	**Lower vascular pedicle**	**Parathyroids**	**Superior laryngeal nerve**	**Inferior laryngeal nerve**	**In presence of blood**
**No. 1**	5	5	5	5	5	2
**No. 2**	5	5	5	5	5	2
**No. 3**	5	4	4	4	4	1

Score 5: very easy; score 4: easy; score 3: normal; score 2: difficult; score 1: very difficult.

## Discussion

MIVAT was demostrated to be a feasible and safe procedure only if selection criteria are strictly observed. During the last decade, indications for MIVAT were revised including 3.5 cm nodules in the maximum diameter and 25 mL thyroid volume [1,2]. Indications were also extended to patients with associated thyroiditis and those with intermediate-risk differentiated thyroid cancer (DTC) rather than those with low risk DTC [2]. After a first scepticism about the procedure by some surgeons, actually, MIVAT represents the first choice in many centres treating thyroid disease. Complications are comparable to the conventional technique [1], but, according to a meta-analysis reported in literature, MIVAT needs longer operative time to be accomplished even if it is superior in terms of immediate postoperative pain and cosmetic results [3-5]. Nevertheless, one restriction of endoscopic or endoscope-assisted surgery is the lack of binocular or stereoscopic vision. Monocular endoscopes give a 2D image that may impair depth perception, hand-eye coordination, and size evaluation. Some studies in other fields of application demonstrated that, although not in a strictly objective way, severe mistakes made during endoscopic procedures reflect a critical misinterpretation of the video image rather than simply technical errors [6].

We are the first to describe the use of the 3D endoscope for MIVAT in a small group of patients due to verify its safety and effectiveness in a preliminary report. The indications and contraindications for surgery were the same as in the 2D MIVAT. Neither complications as hypoparathyroidism nor vocal cord paralysis were observed. Conversion into conventional thyroidectomy or reoperation for hemostasis were never required. Hospital stay after 3D MIVAT was acceptable, not exceeding 24 hours in any case.

Quality of vision was considered optimal by all the users except in the presence of blood in the surgical field corresponding to a darker vision on the screen, as it happens with 2D systems. In contrast to other experiences [7,8], the glasses were still worn without disadvantages when endoscope was not required. Surgeons did not report any side-effects such as fatigue, headache, dizziness, and eye strain during or after surgery.

According to some authors, stereoscopic visualization improves depth perception, anatomical understanding, efficiency of surgical movement, and surgeon confidence. The improvement of second-generation endoscopic stereoscopic systems would probably improve task performance, shorten operative time, and decrease error rate [7,8].

This preliminary study seems to confirm that 3D MIVAT is safe. The aim of the study was not to compare the 3D versus the 2D technology, but to evaluate safety and technical feasibility. A huge number of cases would be necessary to demonstrate whether a statistical difference may exist between 2D MIVAT or 3D MIVAT in terms of complications due to the low incidence of them [1,3,4], while results in terms of pain and cosmetic are expected to be similar. This paper anticipate future studies with larger series comparing 2D and 3D MIVAT according to visualization and advantages in the different steps of the procedure. Furthermore, the cost-benefit relationship is not less important and should be investigated.

## Conclusion

3D MIVAT seems to be safe and effective. A subjective good perception in depth was acknowledged by the involved surgeons without any problem in recognising critical anatomical structures. No complications were observed and operative time was acceptable. Future studies with larger case series are required to determine the role of this device.

## Abbreviations

MIVAT: Minimally invasive video-assisted thyroidectomy; 3D: Three-dimensional; 2D: Two-dimensional.

## Competing interests

The authors declare that they have no competing interests.

## Authors’ contributions

Conception and design: GM, wrote the paper, Provision of study materials or patients: PB, VM, GC, RP, GS, All authors have read and approved the final manuscript.
